# Business Risk Mitigation in the Development Process of New Monoclonal Antibody Drug Conjugates for Cancer Treatment

**DOI:** 10.3390/pharmaceutics15061761

**Published:** 2023-06-18

**Authors:** Balázs Kiss, János Borbély

**Affiliations:** 1 Faculty of Economics, University of Debrecen, 4032 Debrecen, Hungary; kiss.balazs@econ.unideb.hu; 2 BBS Dominus LLC, 4225 Debrecen, Hungary; 3 Doctoral School of Clinical Medicine, University of Debrecen, 4032 Debrecen, Hungary; 4 BBS Biochemicals LLC, 4225 Debrecen, Hungary

**Keywords:** antibody–drug conjugate (ADC), nanomedicine, development, nanoparticle drug conjugate (NPDC), linkage, payload, warhead, business risk, open innovation, collaboration

## Abstract

Recent developments aim to extend the cytotoxic effect and therapeutic window of mAbs by constructing antibody–drug conjugates (ADCs), in which the targeting moiety is the mAb that is linked to a highly toxic drug. According to a report from mid of last year, the global ADCs market accounted for USD 1387 million in 2016 and was worth USD 7.82 billion in 2022. It is estimated to increase in value to USD 13.15 billion by 2030. One of the critical points is the linkage of any substituent to the functional group of the mAb. Increasing the efficacy against cancer cells’ highly cytotoxic molecules (warheads) are connected biologically. The connections are completed by different types of linkers, or there are efforts to add biopolymer-based nanoparticles, including chemotherapeutic agents. Recently, a combination of ADC technology and nanomedicine opened a new pathway. To fulfill the scientific knowledge for this complex development, our aim is to write an overview article that provides a basic introduction to ADC which describes the current and future opportunities in therapeutic areas and markets. Through this approach, we show which development directions are relevant both in terms of therapeutic area and market potential. Opportunities to reduce business risks are presented as new development principles.

## 1. Introduction

Medical (red) biotechnology is currently one of the most rapidly developing industries, with disruptive innovations emerging from time to time. Each decision, such as the development of a drug, has the potential to be worth billions of USD. The pharmaceutical industry is among the most research-intensive industries, with an average new product development (NPD) cycle of 11.9 years [1]. The uncertainty in the industry is determined primarily by its nature, which leads to very long development timelines (averaging approximately 10 years from research to market), very large investments (approximately USD 1–2 billion for new molecules), and extremely stringent regulations for product licensing. For these reasons, it is important to minimize business risks during the early stages of drug development. As antibody–drug conjugates (ADCs) are currently one of the most heavily researched areas in biotechnology, it is certainly worthwhile to consider this area from that perspective.

An example from 2017 illustrates the importance of prudent R&D where the pivotal trial of Bayer’s ADC anetumab ravtansine did not meet its primary endpoint, as the drug failed to improve progression-free survival in mesothelioma patients. This resulted in a decline in the stock prices of Bayer’s collaborators, ImmunoGen and MorphoSys [2].

ADC is a type of targeted therapy that combines the specificity of monoclonal antibodies with the cytotoxicity of a small molecule drug. ADCs are designed to deliver a toxic payload specifically to cancer cells, minimizing damage to healthy cells.

The market for ADC is expected to grow significantly in the coming years, driven by the increasing incidence of cancer, the growing demand for targeted therapies, and the development of new and improved ADC technologies. The cancer type that is most targeted by ADCs is breast cancer, followed by lung and ovarian cancers. The market is divided into segments on the basis of the type of drug, with the largest portion of the market held by microtubule inhibitors, followed by DNA-damaging agents and alkylating agents. Additionally, the market is segmented on the basis of its applications, with the highest market share held by solid tumors, followed by hematological malignancies. In terms of geography, North America holds the largest market share, due to the presence of a large number of biotechnology and pharmaceutical companies in the region, followed by Europe and Asia Pacific.

Companies such as Seattle Genetics, ImmunoGen, and Roche are some of the key players in the ADC market. The activity of the market is shown by the fact that continual acquisitions and mergers are also characteristic of the present times. An excellent example of this is the recently announced news that Pfizer and Seagen have entered into a definitive merger agreement under which Pfizer will acquire Seagen, a global biotechnology company that discovers, develops, and commercializes transformative cancer medicines, for USD 229 in cash per Seagen share for a total enterprise value of USD 43 billion [3]. It is the largest acquisition in biopharma since June of 2019, when AbbVie acquired Allergan for USD 63 billion. Early in 2019, Bristol Myers Squibb executed the largest transaction in industry history with its USD 74 billion purchase of Celgene [4].

It is worth mentioning that ADCs are still a relatively new class of therapeutics, and they are still facing a number of challenges. For example, many ADCs that have been developed so far have not shown significant efficacy in clinical trials [2,5], and many have experienced setbacks during development. Additionally, the cost of ADC therapy is high (e.g., yearly ADC treatment regimen costs~USD 100–500 k [6,7]); production costs also are high [8], which could be a barrier for some patients.

In order to overcome these weaknesses, recent developments are creating new constructions to improve efficacy. A new development introduces the advantages of peptide-drug-conjugated constructions [9] vs. ADCs.

In order to increase the payload, drug molecules are located in a nanoparticle, which is conjugated with the mAb. Those new structures are referred to in the literature as antibody-conjugated nanoparticles (ACNP). It is expected that through this structure, the selectivity and the efficacy are improved. Nanoparticles, e.g., liposomes, that are used as chemotherapeutic agent nanocarriers for doxorubicin (Doxil), are successfully conjugated with mAbs, forming the so-called immunoliposomes [10,11,12,13,14]. Targeting immunoliposomes are in clinical trials [15,16]. Other polymeric nanoparticles based on natural or synthetic biopolymers are conjugated with mAb and are carriers of anticancer drugs, such as as doxorubicin (DOX), paclitaxel (PTX), epirubicine, and cisplatin [17,18,19,20,21]. Many other nanocarriers have been conjugated with mAb, e.g., dendrimers [22], gold nanoparticles [23], and magnetic nanoparticles [24].

## 2. The Market of ADCs

**Figure 1:**
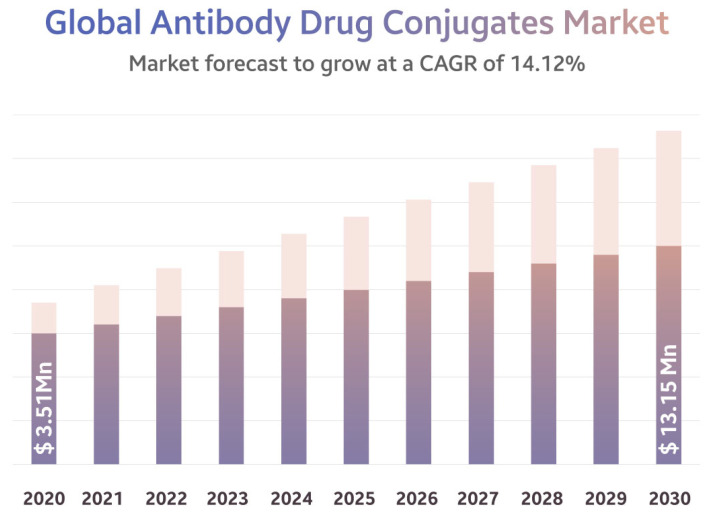
Global ADC market [26]. Quoted with permission from Strategic Market Research. Available online: https://www.globenewswire.com/en/news-release/2022/06/21/2465821/0/en/Antibody-Drug-Conjugate-Market-a-13-15-billion-Industry-by-2030-with-a-CAGR-of-14-12.html (accessed on 21 June 2022).

The mAbs, e.g., Herceptin (Trastuzumab (TZM)), are a class of anticancer agents. Kadcyla represents the gold standard for the treatment of HER2+ breast cancer patients [25]. Recent developments aim to extend the cytotoxic effect and therapeutic window of these type of mAbs by constructing ADCs in which the targeting moiety is the mAb that is linked to a highly toxic drug. As of the end of 2022, there were 14 ADCs marketed, and there are approximately 100 others in clinical trials for different indications. According to a report from the middle of last year, the global ADC market accounted for USD 1387 million in 2016 and was worth USD 3.51 billion in 2020, registering a compound annual growth rate (CAGR) of 14.12%. It is estimated to grow at a rate of 17.08% and will increase in value to USD 13.15–16.4 billion by 2030 [26,27] as Figure 1 shows.

One might inquire as to the significance of ADCs and the reasons for their substantial market growth rate. Let us provide a concise summary of the principal determinants that underlie this phenomenon. Primary drivers stimulating the global market expansion are the following: ADCs are developed to target the cancer cells alone, bypassing the healthy cells. Using this phenomenon, ADCs are used primarily in cancer treatment. According to Eurostat, cancer was responsible for the deaths of approximately 1.2 million people in Europe, which was 26% of the total deaths in the region. According to the CDC, breast cancer is the second most common type of cancer in women in the US and is responsible for nearly 42,000 deaths each year. Thus, with rising cancer cases globally, the need for ADCs will boost the overall market growth. As per UN statistics, globally, the number of people over the age of 65 will rise from 9.3% of the total population in 2020 to 16.0% of the population by 2050. To ensure a good quality of life at this increased average age, more targeted, effective cancer therapies, such as ADCs, will be needed. The increase in the average age of individuals can be explained by their adoption of healthy habits and lifestyles, which has led to greater awareness of these practices [26].

The current market status is as follows: Since the first ADC, Mylotarg^®^ (gemtuzumab ozogamicin), was approved in 2000 by the US Food and Drug Administration (FDA), there have been 14 ADCs that received market approval so far worldwide. As of November 2022, the FDA has approved 13 different ADCs, including Lumoxiti (moxetumomab pasudotox-tdfk), which we consider as an immunotoxin.

The market for ADCs is expected to continue to grow beyond 2026, as novel agents are introduced into clinical practice for several oncology indications [26,27].

Table 1 summarizes the trade name, maker, payload design, and approved indications of these drugs [28]. Currently, there are more than 100 ADCs being developed for clinical use, with the majority intended for the treatment of cancer.

## 3. Nanotechnology in Medical Biotechnology

New developments are in progress to increase the selectivity as well as the efficacy of ADCs. One of hot areas of these types of developments is nanomedicines. Recognizing the importance of a comprehensive understanding of advances in pharmaceutical R&D, we felt it valuable to provide a concise overview of current developments in nanomedicines within the field of medical biotechnology when discussing the development of ADCs. Some examples of the different types of nanomedicines that are being developed and studied for various therapeutic applications can be found below. Each type of nanomedicine has unique properties and advantages that make them suitable for specific therapeutic or diagnostic purposes.

### 3.1. Nanoparticle-Based Drug Delivery

Nanoparticles can be used to encapsulate the drugs and deliver them to specific target sites, such as tumors or inflamed tissues. This approach can improve the efficacy and reduce the side effects of the drugs [29,30].

### 3.2. Gene Therapy

Nanoparticles can be used to deliver genetic material, such as DNA or RNA, into cells for therapeutic purposes. This approach has the potential to treat genetic disorders and some types of cancer [31,32,33].

### 3.3. Photodynamic Therapy

This involves using photosensitizing agents that are activated by light to kill cancer cells or bacteria. Nanoparticles can be used to deliver photosensitizers to target sites and improve the specificity of the treatment [32,34,35].

### 3.4. Immunotherapy

Nanoparticles can be used to deliver immunotherapeutic agents, such as antibodies or cytokines, to target sites to boost the immune system’s response against cancer or other diseases [32,36,37].

### 3.5. Tissue Engineering

Nanofibers and other nanomaterials can be used to create scaffolds for tissue engineering applications, such as repairing damaged tissues or organs [38,39].

### 3.6. Diagnostic Imaging

Nanoparticles can be used as contrast agents for various imaging modalities, such as positron-emission tomography (PET) [40,41], magnetic resonance imaging (MRI) [42,43], or computed tomography (CT) [44] scans, to detect and diagnose diseases.

The nanomedicine market is a rapidly growing sector that includes the development and application of nanotechnology in medicine. According to a market research report by Grand View Research, the global nanomedicine market size was valued at USD 215.0 billion in 2020 and is expected to grow at a CAGR of 13.6% from 2021 to 2028. The report suggests that the increasing prevalence of chronic diseases, such as cancer, diabetes, and cardiovascular diseases, along with the growing demand for personalized medicine, is driving the growth of the nanomedicine market. The report also highlights the rising investments in the research and development of nanomedicines, along with the increasing adoption of nanotechnology in drug delivery and diagnostic applications, as key factors contributing to the growth of the market [45].

When a drug or medical device has a therapeutic effect due to the use of nanomaterials, it is important to understand how the pharmacodynamic and pharmacokinetic responses have been affected by the size of the nanomaterial. The EUON (European Union Observatory for Nanomaterials) has a database with more than 1000 products that use nanomaterials, including 91 healthcare products, such as wound dressings, implants, and liposomal drugs for indications such as cancer, cardiovascular disease, diabetes, and infection. The US Nanomaterial Consumer Products Inventory lists 762 nanotechnology-related products in the fitness and health category, but information on the composition of the nanomaterial is missing for almost half of them.

In the pharmaceutical industry, only a small number of platform technologies are commonly used, according to submissions to the Center for Drug Evaluation and Research (CDER) of the US FDA. Some nanocrystal formulations and liposomes, considered non-biological complex drugs (NBCD), are challenging to manufacture under good manufacturing practice (GMP) conditions and require thorough physicochemical and biopharmaceutical characterization [46].

The nanomedicine market includes several segments, such as nanoscale therapeutic agents, nanodiagnostics, and nanotechnology-based medical devices. The report suggests that the nanoscale therapeutic agents segment held the largest share of the market in 2020, driven by the increasing demand for targeted drug delivery and the development of novel drug delivery systems [45].

Overall, the nanomedicine market is expected to continue its growth trajectory in the coming years, driven by advancements in nanotechnology, increasing demand for personalized medicine, and rising investments in research and development.

## 4. R&D of ADC Technologies

**Figure 2:**
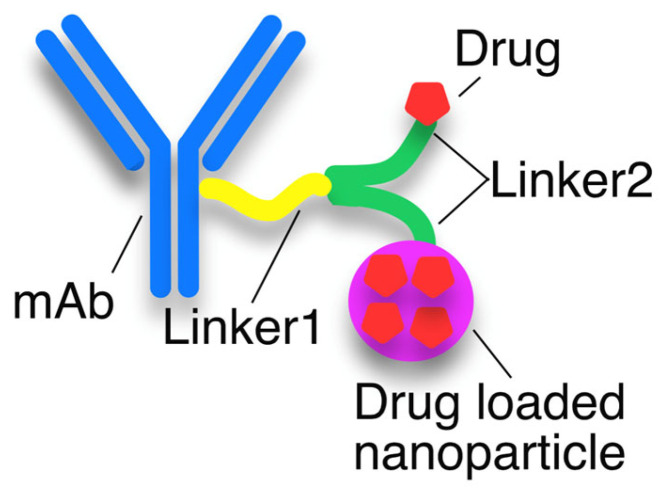
Schematic structure of antibody–drug conjugate (ADC). The functional group (e.g., amino group of Lys/Cys moiety) of mAb (monoclonal antibody). Drug molecules are linked directly to the mAb or are loaded into a nanoparticle linked to the mAb.

**Figure 3:**
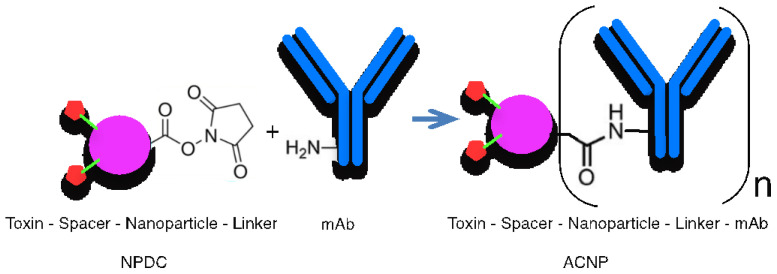
Schematic structure of NPDC and ACNP.

In the new class of drugs, mAbs and mimetics [47,48] can play a dual role. ADCs contain antibodies directly linked to a limited number of highly toxic drug warheads through a linker (Figure 2). However, in the class of ACNP drugs [14,49], a large number of drug molecules are enclosed in a special nanocarrier. The NPDCs serve as a cargo to improve the drug concentration at the tumor site, by passive targeting mechanisms due to their enhanced permeability and retention ability. In order to overcome the limitations due to low drug concentration of the ADCs, the nanoparticle–drug combination is conjugated with the antibody (Figure 3). Thus, the specificity of ACNP is improved by mAb or mimetics, and the higher cargo is provided by the NPDC. In the development process, researchers must take into account the strengths of each of the mentioned drug classes.

The mAbs that are created for the treatment of cancer use various mechanisms to fight the disease, such as antibody-dependent cell-mediated cytotoxicity (ADCC), complement-dependent cytotoxicity (CDC), and alterations in cell signaling. One well-designed drug for targeting HER2 in cancer therapy is TZM. However, clinical data show that many patients with HER2-overexpressing breast cancer do not respond to TZM-based therapies. To address this issue, a new type of biopharmaceutical drug, called ADC, is being developed. ADCs consist of an mAb as a targeting molecule, and a highly cytotoxic drug that is attached to a linker with a functional group, which is then connected to the Lys amine group and Cys thiol group of the mAb [50]. The primary advantage of ADCs is that the cytotoxic drug is specifically delivered to the tumor cell, increasing the efficacy and reducing the side effects due to their limited off-target toxicity [51].

Recently, several drug-loaded nanoparticles have been in development for conjugation with mAb, e.g., functionalized magnetic nanoparticles [52], including trastuzumab-modified gold nanoparticles [53], as potential multimodal agents. It was found that these nanoparticles bind with high specificity to the HER2+ cell lines but not to the HER2- cell lines. Biopolymer-based as well as poly(lactide-co-glycolide) (PLGA) nanocarriers were loaded with the oxalilplatin chemotherapeutic drug and coated with mAb [54]. There is a study which demonstrated that the anti-cancer drug loaded in polymeric micelles or cyclodextrins improved the stability and the solubility of the prodrug, which was then connected to mAb, resulting in high efficacy [55].

Recent strategies for developing new ADCs involve selecting new components for targeting.

- mAb selection: For targeting, biologics, such as mAbs, fragments, and other backbones (e.g., single-chain variable fragment (scFv), affibody, Pentarin, and antibody–cytokine fusion proteins), are used to target HER2 or other antigens. Generally, lysines with free amines are more common than cysteines with disulfides and are not evenly distributed in the antibody [56,57]. However, Genentech is currently testing modified antibodies with engineered cysteines. Antibody mimetics are also the focus of recent research due to their importance. The site specificity/efficacy of the composed drug can be improved by adding a new building block as an antibody mimetic, e.g., single domain antibody [58], nanobody [59], or affibody [60].
- Toxins: There are numerous cytotoxic drugs that can be used as payloads, such as Maytansine (DM1, DM4), Auristatine (MMAE), SN-38, Doxorubicin, and Duocarmycin analogues [61].
- Linker selection: Linkers have a crucial role in the ADC and ACNP constructions, respectively. This part of the construction is responsible for the stability of cargo. The linker must be stable during the circulation in the bloodstream to avoid the leakage of drug molecules. A class of linkers is designed according to bio-orthogonal chemistry [62], which allows cleavage in the microenvironment of cancer cells [63]. Linkers can be either cleavable or non-cleavable, with various types of cleavable linkers, such as chemically labile linkers and enzyme-cleavable linkers (e.g., pH-sensitive linkers, disulfide linkers, peptide linkers, β-glucuronide linkers, and aldehyde tags [64]). Examples of linker platforms include the ImmunoGen Platform, Val-Cit, Disulphide, and Hydrazon [65]. For the nondegradable linkers, the connection of the cytotoxic and the antibody is non-sensitive to proteolytic degradation [66].

In the construction of ADCs, the biomolecule antibody requires precise chemical modification under specific conditions, and the payload capacity is relatively limited. In contrast, the formation of the nanoparticle drug constructs can occur in a less sensitive environment. In the final step of the ACNP formation, the activity of the antibody can be better protected.

There are many developments focusing on how to modify the TZM antibody. In this section, we describe a few steps of a synthesis project for creating a modified version of the trastuzumab antibody as an example. As a first step, lysine residues on the antibody are chosen as the conjugation sites to avoid disturbing the antibody’s structure and function and to maintain its favorable pharmacokinetic properties. Secondly, toxins are selected on the basis of their functional groups, and linkers are chosen for their ability to bind the toxin to the biopolymer and the NPDC to the mAb. Finally, biopolymers are selected on the basis of their solubility in water, ability to self-assemble into nanoparticles of a desired size and distribution, and their functional and steric properties for connection to the mAb.

A new approach to combining mAbs with toxic drugs involves using a biopolymer-based nanoparticle. This type of ADC is composed of a biodegradable biopolymer nanoparticle that contains a highly cytotoxic payload and is decorated with the targeting TZM antibody on the surface. The synthetic route for creating this ADC begins with the creation of a drug/toxin biopolymer conjugate that is coupled through a spacer (PDC). The biopolymer (Poly_1) is capable of self-assembling, and by adding Biopolymer_2 (Poly_2), a nanoparticle containing the drug molecule is obtained (NPDC). The functional groups of NPDC are then modified with a maleimide-containing linker (e.g., SMCC) that can react with lysine groups of the TZM antibody.

This proposed method has potential advantages over other ADCs because the NPDC can be precisely prefabricated [67,68], and in the final step, it can be conjugated to the mAb, resulting in the appropriate ACNP (Figure 3). It is suspected that these constructions have the benefits of highly targeting cancer cells due to their antibody content, as well as a high efficacy due to the high payload carried by the conjugated nanoparticles.

This new structure will allow for a combination of immune and chemotherapeutic nanomedicine, resulting in overall higher anticancer efficacy.

For the class of antibody-functionalized lipid-based nanoparticles or solid–liquid particles, in the first step, different terminal groups, such as amino, carboxyl, maleimide, or NHS, are formed [69], then they are conjugated with the appropriate mAb.

New elements, such as nanoparticles (e.g., dendrimers, PLGA, and polymer-based ADCs), are being explored in ADC development. The Mersena (MA) technology uses a polyacetal polymer-based platform for creating ADCs.

In the field of ADCs, the technology of “cleavable linker” currently has the largest and most robust market share. Cleavable linkers are advantageous because they offer more varied applications compared with non-cleavable linkers due to their ability to use different mechanisms to act on disease sites. In May 2022, Aptamer and PinotBio entered into a collaborative effort to develop Optimer-drug conjugates as an alternative format for ADCs to target four specific non-blood-based cancer targets, namely Nectin-4, Tissue Factor, CEACAM5, and CD73. These biomarkers have the potential to target solid tumors, which have a poor response to chemotherapy. This could lead to the development of therapeutics with a smaller size that would allow for greater penetration of the tumor compared with standard antibody-based ADCs [70,71].

In terms of applications, “Breast Cancer” has the highest market share for ADCs. The number of breast cancer cases has increased dramatically over the years, with the World Health Organization reporting nearly 19.3 million cases in 2020, almost doubled from the 10 million cases reported in 2000. This increase in breast cancer cases has made it the most prevalent form of cancer, surpassing lung cancer. Consequently, ADCs are being used as a viable treatment option, which has increased the market size for ADCs.

Geographically, “North America” holds the largest market share for ADCs. Most of the development in the field of ADCs is conducted in North America, including research and development and clinical trials. The presence of major pharmaceutical companies based in the United States, such as Johnson & Johnson, Pfizer, Gilead Sciences, and Abbott International, has significantly contributed to the revenue share of the region. The American Cancer Society has predicted 1.9 million cancer cases and 609,000 deaths in the region in 2022, which is likely to boost the market for ADCs [26].

Some recent development examples in the market are mentioned below. In June 2022, ADC Therapeutics was in the process of adding its second ADC to the company’s product portfolio, with the introduction of camidanlumab tesirine. This drug has shown promising results in Phase 2 of clinical trials, and the company is likely to seek approval from the US Food and Drug Administration (FDA) to use it as a treatment option for Hodgkin’s lymphoma [72].

Also in June 2022, Spirea Limited announced that it had received GBP 2.4 million in funding from investors to develop highly specialized ADCs for treating solid tumors. Their technology allows for a higher drug-to-antibody ratio, which means that more of the drug can be delivered to the affected area to eliminate cancer cells. This could lead to more effective cancer treatments [73].

In May 2022, the FDA approved the use of fam-trastuzumab deruxtecan-nxki (Enhertu^®^) for the treatment of metastatic HER2-positive breast cancer. The drug was developed by Daiichi Sankyo and AstraZeneca [74].

## 5. Challenges and Business Risks in R&D of ADCs

### 5.1. Risks for R&D, Limits, and Failures

For more than 100 years, the concept of ADCs has existed, but there are still only a limited number of varieties available on the market. Most of the varieties under research are still in the early stages of development. The main reason for this is that the development of ADC drugs is a challenging process, with high technical barriers. ADC drugs require multiple steps to become effective after entering the body, and each step presents significant technical difficulties that must be overcome.

The primary ethical concern regarding medical science and technology is often the potential risk involved, especially for new and unfamiliar technologies. While risk and risk–benefit analyses are only one aspect of ethical oversight, they are often used interchangeably in ethical review and risk assessment. This is because both the Common Rule and FDA emphasize the importance of minimizing risk for human subjects and require the local Institutional Review Boards (IRBs) to consider the risk–benefit analysis when making decisions about proposed research. In the case of ADCs, toxicological analyses are typically the first thing that comes to mind when assessing risk, as they are critical for evaluating the safety of these molecules. There is a significant body of literature on toxicological risk analysis for ADCs, and many new methodologies have been developed and published [75,76,77]. However, business risk methodologies for the development of these molecules have been less discussed. Developing an ADC involves significant technological challenges and a complex development process that can be very expensive. Therefore, the business risk associated with developing an ADC is very high.

In general, the high rates of drug development failures can be attributed to various reasons, including:

- Unreliable published data;
- Biopharmaceutical issues, such as suboptimal pharmacokinetics;
- Poorly predictive preclinical models used in discovery research and preclinical testing;
- The concept of target-based drug discovery, which involves complex target selection, competition for proprietary targets, and the validation process;
- Complexities of clinical trials, particularly in treating chronic diseases, along with increasing demands from regulatory authorities and payers.

Smaller organizations lacking the know-how and resources of larger organizations, leading to lower probability of technical and regulatory success from Phase I to submission [78,79].

While ADCs hold great promise for the treatment of cancer and other diseases, there are several challenges and business risks associated with their development. Some of these challenges and risks include:

- Complex Manufacturing: ADCs are complex molecules that require precise conjugation of the antibody and the cytotoxic agent. The manufacturing process can be challenging and time-consuming, and any variability in the manufacturing process can affect the quality and efficacy of the final product.
- Regulatory Challenges: ADCs are subject to strict regulatory oversight, and the approval process can be lengthy and expensive. Regulators require extensive data on the safety and efficacy of ADCs, including data on the pharmacokinetics, pharmacodynamics, and toxicology of the drug.
- Target Selection: Choosing the right target for an ADC is critical for its success. If the target is not expressed on the tumor cells, or if it is expressed on normal cells, the ADC may not be effective or may cause off-target toxicity [80,81,82].
- Resistance: As with any cancer therapy, the development of resistance is a significant challenge for ADCs. Cancer cells can develop resistance to the antibody, the cytotoxic agent, or both, rendering the ADC ineffective.
- Intellectual Property: ADC development involves complex intellectual property issues, including patenting of the antibody, the linker, and the cytotoxic agent. Companies must navigate these issues carefully to avoid infringement and protect their intellectual property.
- Cost: Developing ADCs can be extremely expensive, with high costs associated with manufacturing, clinical trials, and regulatory approval. There is also significant competition in the market, which can drive down prices and limit profitability.

The characterization of nanotechnology-related products requires a combination of different techniques to understand their physicochemical features and how these impact efficacy and product safety. From a regulatory standpoint, assessment of the environmental toxicity and effects on occupational health are required for the raw materials (excipients), while drug products and medical devices follow their own framework, with a greater emphasis on the therapeutic applications [83].

Pharmaceutical companies are currently facing significant barriers to entering the emerging nanomedicine market, with recent trends in the European regulatory landscape indicating increased restrictions and a narrower field of competitors. However, a growing knowledge base and a rising number of drug products and medical devices in the market offer new opportunities for the industry [83].

The European Medicines Agency (EMA) has established a framework for evaluating nanomedicines, which includes the following principles:

- The evaluation of any nanomedicine should be based on established principles of benefit/risk analysis, rather than solely on the basis of the technology itself (including the use of Risk Management Plans and Environmental Risk Assessment) [77,83].
- Specialized multidisciplinary expertise is required, with a group of mixed academia and regulatory experts pooling their knowledge of quality, safety, and kinetics to support evaluation and formulate guidelines [83].
- Close cooperation with other scientific committees (such as the Scientific Committee on Emerging and Newly Identified Health Risks and the European Food Safety Authority), networks (such as the Nanotechnology Knowledge Base and the European Technology Platform for Nanomedicine), and the European Commission [83].
- International cooperation, with EMA chairing an international expert group that includes the US FDA, Japan MHLW, Health Canada, and TGA Australia.
- Transparent dialogue with stakeholders [83,84].

### 5.2. Case Studies

In 2012, the FDA approvals were reviewed, and the most probable reasons for failures in Phase II and Phase III clinical development were found to be lack of efficacy (56%), safety issues (28%), changing strategies (7%), commercial reasons (5%), and operational challenges (5%). These results were confirmed by a second analysis of 142 drug R&D projects of AstraZeneca, which found that preclinical and Phase I projects failed primarily failed due to safety reasons, whereas projects failing in Phases II and III commonly lacked efficacy [78].

In the pharmaceutical research and development (R&D) process, discovery research takes 4.5 years, preclinical testing lasts for 1 year, and the three clinical development phases require 1.5, 2.5, and 2.5 years, respectively, with an additional 18 months from submission to launch. Basic research and post-approval Phase IV trials must also be considered in the overall R&D time. There are two additional findings when reviewing drug R&D timelines. First, clinical development takes longer than in the past. Second, the average time for FDA review and approval has decreased significantly since the Prescription Drug User Fee Act (PDUFA) was enacted, potentially due to fast-track status or accelerated approvals.

The long overall time of pharmaceutical R&D impacts on total R&D costs increases the risk of industry competition and raises the uncertainty of generic competition. This is due to the capitalization of R&D costs, which increases overall expenditures, the risk of competition, which reduces the chance to be first-to-market, and the commercial success of a drug candidate. Additionally, the effective date of generic competition can influence the ROI of a new drug by reducing the commercially usable patent term.

At present, the duration of the clinical trials is longer due to the COVID-19 pandemic because there are many vaccine candidates in development which are requiring many resources [78].

The biotechnology industry faces the challenge of covering the entire R&D process and clinical costs within a limited timeframe, typically no more than 10 years and sometimes as little as 5 years. To address this challenge, many companies are exploring ways to shorten the development process. One approach is to use open innovation, which involves collaborating with external partners and outsourcing R&D activities instead of relying solely on in-house resources. By leveraging the expertise of external partners, companies can avoid the time and cost of developing new skills internally. This strategy can help accelerate the development process and potentially reduce costs. All of the following factors can contribute to a decrease in R&D efficiency in the pharmaceutical industry. An inadequate number of projects in early R&D phases can lead to a lack of diversity in a company’s pipeline and limit the potential for new drug discoveries. More technically complex research for new drug targets and subsequent preclinical and clinical studies can increase the time and resources required for R&D, which can negatively impact efficiency.

The burden for approval and reimbursement of new molecular entities (NMEs) can also be high, particularly in view of the already-approved drugs in the market. This can lead to a more risk-averse approach from both regulators and society, which may result in longer approval timelines and a lower likelihood of success for new drugs.

Mergers and acquisitions (M&As) can have a negative effect on R&D efficiency as well, particularly if the integration of the two companies results in the duplication of efforts and resources. Similarly, the decreasing number of research-based pharmaceutical companies taking the financial risk of drug R&D can limit competition and innovation in the industry.

Licensing, co-development, or joint ventures can have a negative effect on clinical development and approval durations if there are disagreements or delays in decision-making and resource allocation between the partnering companies. Overall, a combination of these factors can contribute to the challenges facing the pharmaceutical industry in maintaining R&D efficiency and productivity [78].

### 5.3. New Forms for R&D

It is encouraging to see that more pharmaceutical companies are recognizing the need to make changes to their R&D ecosystems in order to improve their R&D efficiencies. The process changes that these companies are making can help them to manage better their R&D activities and reduce costs while also expanding their competence and technology base and strengthening their innovation potential.

One way that companies are creating growth options is through M&As, which can provide access to new technologies and pipelines and expand their market reach [85]. Restructuring R&D into smaller, more manageable units, similar to those found in biotechnology companies, can also help to improve the efficiency and focus on specific therapeutic areas or technologies.

Outsourcing and virtual R&D are increasingly being used by pharmaceutical companies to reduce R&D costs, particularly in areas such as preclinical and clinical development, where third-party service providers can provide cost-efficient solutions. Companies are also widening their competence field by expanding collaborations and research partnerships, which can provide access to new technologies, scientific expertise, and drug candidates in all phases of development.

Venture capital investments are being used to strengthen innovation potential, particularly in the area of early-stage drug discovery, where startups and emerging companies are developing new technologies and drug candidates. Lastly, some companies are using the power of the crowd to broaden their knowledge base, for example, by crowdsourcing ideas and solutions through open innovation platforms.

Overall, the process changes being made by pharmaceutical companies reflect a recognition of the need to adapt to the changing landscape of R&D and to adopt more agile, innovative approaches to improve R&D efficiencies and productivity [78].

The concept of Open Innovation has garnered increasing interest in the past 20 years, particularly since Henry Chesbrough introduced the term in 2003, which led to the development of a new field of knowledge. However, despite the extensive research, there is no standardized, all-encompassing theory of Open Innovation. Instead, there exists a range of models that address different aspects and are applicable to specific contexts and industries. One of the industries that has adopted Open Innovation is the pharmaceutical industry. The pharmaceutical industry’s move towards Open Innovation has unique features, such as the need to address the current productivity crisis as a driver for change and the R&D-intensive nature of the industry [86].

Due to the high complexity of the biopharmaceutical industry, outsourcing, “slicing up” of tasks, and collaboration with other companies are more likely to occur. Another characteristic of the sector is that the development and production of a biologically active ingredient involves thousands of steps, resulting in a long value chain. At almost every step, management must decide whether to keep the given activity in-house or to outsource it.

In modern biotechnology projects and programs, there is a trend towards project network organizations (PNOs) as opposed to project-based firms (PBFs), which have become increasingly important contexts for interorganizational project cooperation. Due to organizational specialization, PNOs have become general organizational forms that combine the coordination ability of PBFs with the resource richness of networks. PNOs connect legally independent but often operationally interdependent individuals and organizations in the form of strategically coordinated project teams and flexible partner groups that persist beyond individual projects. On the basis of empirical review, PNOs have outstanding characteristics in complex product and system development, research, open innovation, and international development. Differences between the two structures can be related to the variety and connectivity of the projects, the degree of specialization, and the geographical concentration of the resources [87].

## 6. Conclusions

A review of social research in the healthcare industry suggests that there will be a significant demand for effective oncology solutions in the near future, indicating a vast potential market and high risks for medical biotechnology, including ADCs. Market analyses, forecasts, and acquisitions clearly demonstrate this potential. We are on the cusp of a major breakthrough, with only 14 approved ADC products and more than 100 in clinical trials, a number that is set to increase rapidly. Regulatory and clinical acceptance for ADCs is also improving. However, there are technological challenges that make the situation difficult, which could be addressed by the NPDC route as a technological option.

Given the recent excitement surrounding the potential of artificial intelligence (AI) in drug discovery and development, particularly driven by advancements in machine learning and its role in the competitive race for the next blockbuster drug, it is expected that pharmaceutical companies will leverage these computer-based platforms for the development of the next-generation ADCs [88].

Moreover, the potential of ADCs as a therapeutic strategy extends beyond cancer and is substantial. Research is already being conducted on the use of ADCs for the treatment of non-oncological conditions, such as autoimmune and cardiovascular diseases, diabetes, and antimicrobial infections [89].

Since biopharma R&D is highly capital-intensive, it is crucial to identify business risks as early as possible and minimize them where possible. New development principles, such as open innovation, new assembly structures, and new structures for collaboration, offer an opportunity to achieve this.

## Figures and Tables

**Table 1:** FDA-approved ADCs as of November of 2022 [28].

ADC Drug	Maker	Disease Indication	Payload/Payload Class	Target	mAb	Linker	Approval Year
Mirvetuximab soravtansine	ImmunoGen	Platinum-resistant ovarian cancer	Maytansinoid DM4	FRα	IgG1	/	2022
Tisotumab vedotin-tftv	Seagen Inc	Recurrent or metastatic cervical cancer	MMAE/auristatin	Tissue factor	IgG1	Enzyme-cleavable	2021
Loncastuximab tesirine-lpyl	ADC Therapeutics	Large B-cell lymphoma	SG3199/PBD dimer	CD19	IgG1	Enzyme-cleavable	2021
Belantamab mafodotin-blmf	GlaxoSmithKline (GSK)	Adult patients with relapsed or refractory multiple myeloma	MMAF/auristatin	BCMA	IgG1	Non-cleavable	2020, withdrawn on 22 November, 2022
Sacituzumab govitecan	Immunomedics	Adult patients with metastatic triple-negative breast cancer (mTNBC) who have received at least two prior therapies for patients with relapsed or refractory metastatic disease	SN-38/camptothecin	TROP2	IgG1	Acid-cleavable	2020
Trastuzumab deruxtecan	AstraZeneca/Daiichi Sankyo	Adult patients with unresectable or metastatic HER2-positive breast cancer who have received two or more prior anti-HER2 based regimens	DXd/camptothecin	HER2	IgG1	Enzyme-cleavable	2019
Enfortumab vedotin	Astellas/Seagen Genetics	Adult patients with locally advanced or metastatic urothelial cancer who have received a PD-1 or PD-L1 inhibitor and a Pt-containing therapy	MMAE/auristatin	Nectin4	IgG1	Enzyme-cleavable	2019
Polatuzumab vedotin-piiq	Genentech, Roche	Relapsed or refractory (R/R) diffuse large B-cell lymphoma (DLBCL)	MMAE/auristatin	CD79	IgG1	Enzyme-cleavable	2019
Moxetumomab pasudotox	Astrazeneca	Adults with relapsed or refractory hairy cell leukemia (HCL)	PE38 (Pseudotox)	CD22	IgG1	Cleavable	2018
Inotuzumab ozogamicin	Pfizer/Wyeth	Relapsed or refractory CD22-positive B-cell precursor acute lymphoblastic leukemia	Ozogamicin/calicheamicin	CD22	IgG4	Acid-cleavable	2017
Trastuzumab emtansine	Genentech, Roche	HER2-positive metastatic breast cancer (mBC) following treatment with trastuzumab and a maytansinoid	DM1/maytansinoid	HER2	IgG1	Nnon-cleavable	2013
Brentuximab vedotin	Seagen Genetics, Millennium/Takeda	Relapsed HL and relapsed sALCL	MMAE/auristatin	CD30	IgG1	Enzyme-cleavable	2011
Gemtuzumab ozogamicin	Pfizer/Wyeth	Relapsed acute myelogenous leukemia (AML)	Ozogamicin/calicheamicin	CD33	IgG4	Acid-cleavable	2017; 2000

## Data Availability

Not applicable.

## Abbreviations

ADC	Antibody–Drug Conjugate
mAb	Monoclonal Antibody
HER	Human Epidermal Growth Factor
TZM	Trastuzumab
CAGR	Compound Annual Growth Rate
ADCC	Antibody-Dependent Cell-Mediated Cytotoxicity
CDC	Complement-Dependent Cytotoxicity
MRI	Magnetic Resonance Imaging
CT	Computer Tomography
ACNP	Antibody-Conjugated Nanoparticle
NPDC	Nanoparticle–Drug Conjugate
PDUFA	Prescription Drug User Fee Act
ROI	Return of Investment
NME	New Molecular Entity
M&A	Mergers and Acquisitions
PBF	Project-Based Firm
PNO	Project Network Organization
